# Transposase interaction with the β sliding clamp: effects on insertion sequence proliferation and transposition rate

**DOI:** 10.1038/srep13329

**Published:** 2015-08-26

**Authors:** Héctor Díaz-Maldonado, Manuel J. Gómez, Mercedes Moreno-Paz, Patxi San Martín-Úriz, Ricardo Amils, Víctor Parro, Francisco J. López de Saro

**Affiliations:** 1 Centro de Astrobiología (INTA-CSIC), Ctra. de Ajalvir, Km. 4, Torrejón de Ardoz, 28850 Madrid, Spain; 2 Centro de Biología Molecular Severo Ochoa (UAM-CSIC), 28049 Madrid, Spain

## Abstract

Insertion sequences (ISs) are ubiquitous and abundant mobile genetic elements in prokaryotic genomes. ISs often encode only one protein, the transposase, which catalyzes their transposition. Recent studies have shown that transposases of many different IS families interact with the β sliding clamp, a DNA replication factor of the host. However, it was unclear to what extent this interaction limits or favors the ability of ISs to colonize a chromosome from a phylogenetically-distant organism, or if the strength of this interaction affects the transposition rate. Here we describe the proliferation of a member of the IS*1634* family in *Acidiphilium* over ~600 generations of cultured growth. We demonstrate that the purified transposase binds to the β sliding clamp of *Acidiphilium*, *Leptospirillum* and *E. coli*. Further, we also demonstrate that the *Acidiphilium* IS*1634* transposase binds to the archaeal sliding clamp (PCNA) from *Methanosarcina*, and that the transposase encoded by *Methanosarcina* IS*1634* binds to *Acidiphilium* β. Finally, we demonstrate that increasing the strength of the interaction between β and transposase results in a higher transposition rate *in vivo*. Our results suggest that the interaction could determine the potential of ISs to be mobilized in bacterial populations and also their ability to proliferate within chromosomes.

Insertion sequences (ISs) are the simplest mobile genetic elements as they often contain just a single gene encoding the transposase required for transposition. ISs are ubiquitous in bacterial genomes, where they play a key role in genome evolution by promoting chromosomal rearrangements and gene inactivation and mobilization^1^,^2^. A characteristic feature of ISs is their ability to cross species barriers and transpose actively in new hosts, which makes ISs essential players in the process of horizontal gene transfer (HGT). IS-mediated HGT has been documented extensively among habitat-sharing organisms for Bacteria and Archaea^3^,^4^. ISs have often been observed flanking recently-incorporated foreign DNA in genomic islands^5^, or in smaller gene clusters in, for example, pathogens^6^, extremophiles^7^, or endosymbionts^8^. However, the invasion of a new host relies on the compatibility of the IS with the new molecular environment: the transposase gene has to be transcribed and translated, and any interactions of the transposase with host functions ought to be maintained^9^. Indeed, since most ISs do not confer a direct selective advantage to the host, IS persistence in populations implies that they must be constantly imported into genomes by HGT^2^,^3^,^10^. It could be predicted that an active IS has a short time-window to proliferate in a chromosome before inactivating mutations accumulate^11^,^12^,^13^,^14^. ISs have been classified into a number of families depending on their organization, terminal inverted repeats and mechanism of transposition^1^. While some IS families are widely distributed in Bacteria and Archaea, others seem restricted to specific groups. Similarly, some IS families can proliferate explosively in a given bacterial lineage and account for a large fraction of the genome, while others are always found in one or few copies in the chromosome^15^. IS abundance has been correlated with lifestyle or with episodes of rapid evolution, such as the colonization of new ecological niches^16^,^17^,^18^.

Although ISs are highly autonomous, there are several examples of interaction with host enzymes and processes, especially chromosomal replication (for a review, see Ref. 1). The association of transposition to replication was first observed for IS*10*, which is activated by the passing of the replication fork^19^. Recently it has been demonstrated that transposases of various IS families interact with the host replication machinery via the β sliding clamp, an essential factor of DNA replication and repair^20^,^21^,^22^. β is a homodimeric protein that encircles DNA and serves as an anchoring platform for diverse enzymes. In addition to conferring processivity to DNA polymerases, β targets enzymes to the chromosome and the replication fork. These enzymes, including the transposases, often contain a short β-binding motif at their C-terminal end, consisting of the consensus sequence Q-L-x-L-F^21^,^23^,^24^. The fact that transposases with very different transposition pathways and mechanisms bind to β suggests convergent evolution for a critical contact with the host^21^. The ability of a transposase to bind to β could possibly determine its fate during the invasion of a new host. If binding is weak, the transposase may not have a chance to compete with the many other host factors (or other transposases) that also bind to β, transposition could fail and the IS would become inactivated by mutation. Alternatively, if the transposase binds strongly to β, competition with host enzymes would likely interfere with essential host functions and be detrimental to the cell. Depending on the role of β in transposition, a strong interaction could either favor or block transposase activity.

The goal of this study was to determine whether the interaction of transposases with β is a possible requirement that limits IS movement across species, and whether the strength of the interaction could influence the frequency of transposition events. We analyzed the dynamics of IS populations during 600 generations in a recently-isolated acidophilic bacterium, *Acidiphilium* sp. PM, and identified those ISs that had changed in copy number during that period of time. We detected proliferation of a member of the IS*1634* family, a commonly observed IS element in the genome of acidophiles. We demonstrated that the IS*1634* transposase can physically interact with the *Acidiphilium* β sliding clamp and with β from other acidophiles. We then tested the ability of the *Acidiphilium* IS*1634* family member to proliferate in *E. coli*, an organism in which this IS family has never been detected, and obtained direct evidence suggesting that the affinity of the transposase for β could directly determine transposition rate. Finally, we also found that peptides containing the β binding motif of the transposase encoded by the *Acidiphilium* IS*1634* can bind to an archaeal processivity factor, PCNA, from *Methanosarcina*, and that peptides derived from a *Methanosarcina* IS*1634* transposase can bind to the *Acidiphilium* β sliding clamp.

## Results

### A member of the IS*1634* family is active in *Acidiphilium* sp. PM, and its transposase contains a β-binding motif

The sequencing of the genome of *Acidiphilium* sp. PM, an acidophilic α-proteobacterium isolated from the Tinto river in the south of Spain, revealed that up to 2.4% of its chromosome is composed of repeated regions^25^. 85 ORFs related to insertion sequences (transposases and associated factors) were detected and classified by comparative analysis against the Pfam and ISfinder databases (See Ref. 21 for details). Although some of these ORFs corresponded to full-size transposases or associated proteins, many of them were likely inactivated remnants and gene fragments that are no longer capable of transposition. In order to identify active transposable elements, we performed a long-term cultivation of *Acidiphilium* sp. PM. and analyzed changes in IS abundance that had become fixed in the population at the end of the experiment. The sequenced strain was used to start a culture which was then grown for ~600 generations over four years (See Methods). To identify changes in the number of ISs in the population, we used an oligonucleotide microarray consisting of probes representing 1,358 genes associated to ISs, phage and plasmid mobility functions present in the genomes of selected acidophilic organisms, including *Acidiphilium* sp. PM (see Methods). Although this methodology would not detect transposition events that did not change IS copy number in the chromosome (i.e., conservative transposition) or chromosomal rearrangements, we expected that it would detect IS proliferation or loss.

**Figure 1:**
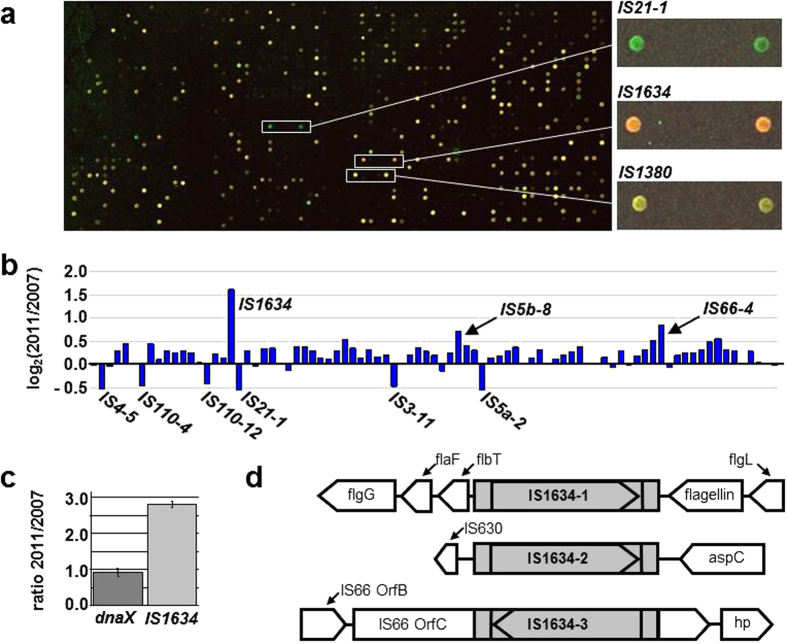
Identification and quantification of IS changes in a 600-generation culture of *Acidiphilium* sp. PM. (**a**) Oligonucleotide microarray to identify and quantify changes in ISs copy number in chromosomes. Three examples of are shown: for a IS that was lost (IS*21*-1), increased in copy number (IS*1634*-1), and remained without change (IS*1380*-1). (**b**) Plot of the relative change (log_2_[‘2011’/‘2007’]) of transposases (or fragments) observed with the oligonucleotide microarray. Each bar represents the average change for each transposase, calculated from the fluorescence of two different oligos replicated three times in each microarray. The microarrays were assayed three independent times. Relevant ISs are indicated (see Table S1 for the full list). (**c**) qPCR experiment to quantify the relative change of IS*1634* from the ‘2007’ to the ‘2011’ culture. DnaX, encoding the γ subunit of DNA polymerase III, and present in single copy in the chromosome, was chosen as a reference. (**d**) Inverse PCR sequencing showed the genomic context of IS*1634*-1 in the ‘2007’ culture and the two additional copies present in the ‘2011’ culture. IS*1634*-1 and 2 were located in the contig NZ_AFPR01000455 (10547 bp), while IS*1634*-3 was located in contig NZ_AFPR01000454 (3091 bp). ‘hp’, hypothetical protein. The inverted repeats were ‘ACTAGT’ for IS*1634*-1, ‘TCTAAA’ for IS*1634*-2, and ‘GATAGA’ for IS*1634*-3.

DNA obtained from the founder strain (‘2007’) and from the 4-year-old population (‘2011’) were differentially labelled with fluorescent dyes Cy3 and Cy5 and hybridized together against the microarray (Fig. 1a). We observed changes (log_2_[‘2011’/‘2007’]>0.5) in nine elements (Fig. 1b and Table S1). Changes implied the apparent loss of the IS ORF in six cases (IS*4*-5, IS*110*-4, IS*110*-12, IS*21*-1, IS*3*-11, IS*5a*-2), and a small gain in copy number in three cases (IS*1634*-1, IS*5b*-8 and IS*66*-4).

A member of the IS1634 family, IS*1634*-1, present in single copy in the ‘2007’ culture, showed an increase to three copies in the ‘2011’ culture, and we focused on this element for the rest of the study. IS*1634*-1 in the sequenced ‘2007’ culture (Fig. S1, Accession number: WP_007423974) could be a recent insertion in the chromosome, as suggested by its anomalous %GC and GC skew (Fig. S2). The copy number increase was confirmed by qPCR, inverse PCR, and sequencing (Fig. 1c,d). Our analysis of the insertion sites of the new copies (IS*1634*-2 and IS*1634*-3) revealed the characteristic inverted repeats generated by true transposition events, but did not detect obvious adaptive benefits for the evolving culture of *Acidiphilium*.

**Figure 2:**
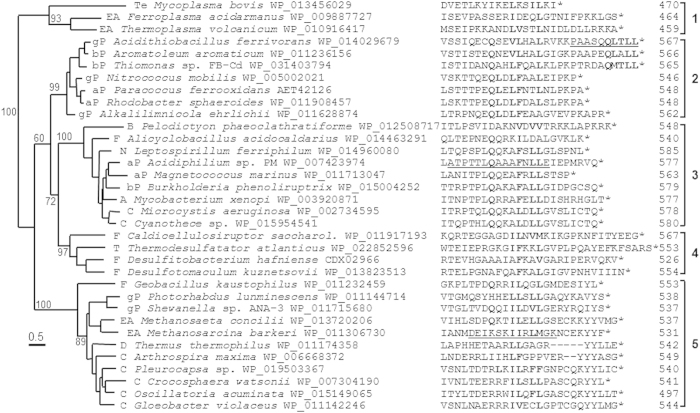
Unrooted similarity tree of IS*1634* transposases and alignment of C-terminal region. IS*1634* transposase sequences were obtained from a previous study^21^, in which 2,216 genomes downloaded from the National Centre for Biotechnology Information (NCBI) Genome database on October 2012, were systematically scanned to identify and classify ISs. The collection of IS*1634* transposase sequences was made non-redundant at a sequence similarity level of 90% and by choosing one representative sequence per genus. The tree was computed with PhyML using a JTT model and a bootstrap of 500 replicates, and the results visualized with Seaview^61^. Bootstrap values are shown for the main branches. The analysis reveals five distinct groups of IS*1634* transposases. The code preceding each organism name is as follows: aP, bP, gP and dP stand for α, β, γ and δ Proteobacteria, respectively; F, Firmicutes; Te, Tenericutes; N, Nitrospira; C, Cyanobacteria; T, Thermodesulfobacteria; D, *Deinococcus*-*Thermus*; A, Actinobacteria; EA, Euryarchaeota. For each transposase the sequence of the C-terminus of the protein is presented and residues involved in the putative β-binding motif are aligned and in bold. Underlined sequences are those of peptides used for biochemical analysis in this work.

The IS*1634* family is a poorly characterized family of insertion sequences related to IS*4*, IS*H3* and IS*701*^26^. A similarity tree for the transposases encoded by IS*1634* members (Fig. 2) shows that this IS family can be found in Euryarchaea and in numerous groups of Bacteria. Interestingly, IS*1634* appears in organisms that share acidic, heavy-metal rich environments, and is present, among others, in the chromosomes of most species of *Leptospirillum* (Nitrospira), *Acidithiobacillus* (γ-proteobacteria) and *Acidiphilium* (α-proteobacteria), as well as in *Paracoccus ferrooxidans* (α-proteobacteria), *Ferroplasma acidarmanus* (Euryarchaea), *Alicyclobacillus acidocaldarius* (Firmicutes), *Thiomonas* sp. (β-proteobacteria), and *Desulfobacca acetoxidans* (δ-proteobacteria) (Fig. 2). IS-sharing has been observed before for co-habiting organisms^3^.

The interaction of transposases with the host β sliding clamp occurs almost always at the C-terminus of the protein via a short sequence with a weak consensus sequence (Q_1_-L_2_-x-L_4_-F_5_)^21^. A search of the β-binding motif among IS*1634* transposases shows that a putative motif can be found at the C-terminus of these proteins (Fig. 2). Despite high variation in sequence context, the alignment of this region shows conservation of the Gln at position 1 of the putative β-binding motif plus conservation of hydrophobic or aromatic residues at positions 4 and 5, suggesting a possible interaction of IS*1634* transposases with the host β sliding clamp via this region.

### The IS*1634* transposase from *Acidiphilium* can bind β from diverse organisms

**Figure 3:**
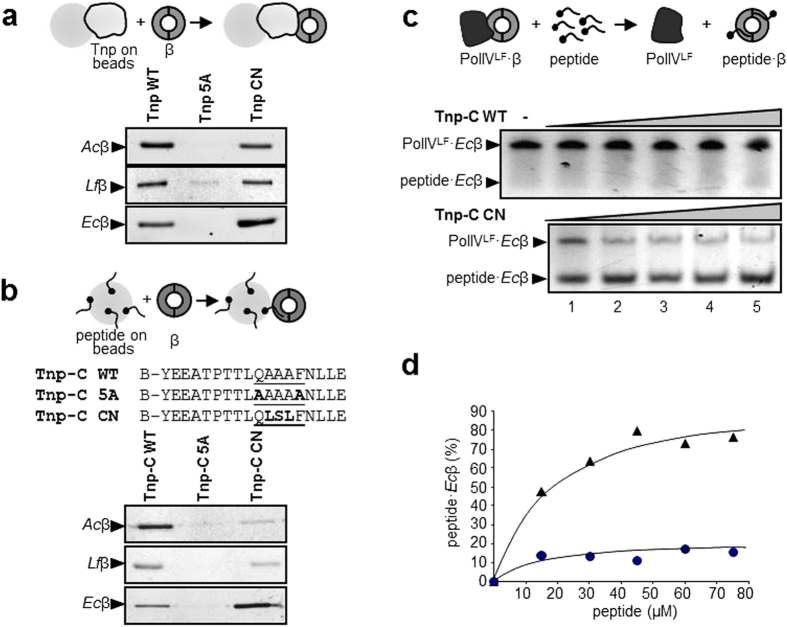
Interaction between IS*1634* transposases and sliding clamps. (**a**) Binding assay of wild-type (WT) and mutants 5A and CN of *Acidiphilium* IS*1634* Tnp to labelled β from, top to bottom, *Acidiphilium*, *Leptospirillum* and *Escherichia coli*. Tnp was covalently coupled to tosyl-activated magnetic beads and any β retained analyzed by SDS-PAGE. (**b**) Sequence and binding assay of peptides derived from C-terminal sequences of wild-type and mutants 5A and CN of *Acidiphilium* IS*1634* Tnp. The amino acids putatively comprising the β-binding motif are underlined, and those changed from the WT sequence are in bold type; B, biotin; the sequence of the C-terminus of *Acidiphilium* IS1634 Tnp begins at residue 4 of the synthetic peptide, see Fig. 2. The N-terminally biotinylated peptides were bound to streptavidin beads and assayed with labelled β from *Acidiphilium*, *Leptospirillum* and *Escherichia coli*. β retained was analyzed by SDS-PAGE. (**c**) Competition assay of complexes of *E. coli* β bound to the C-terminal domain (‘little finger’, LF) of DNA polymerase IV (PolIV^LF^). The WT (top panel) and CN (bottom panel) peptides derived from *Acidiphilium* IS*1634* Tnp were used to disrupt PolIV^LF^·*Ec*β complexes. Concentration of peptides is as follows for lanes 1–5: 15, 30, 45, 60, 75 μM. See Ref. 21 for a detailed description of the methodology. (**d**) Quantification of PolIV^LF^·*Ec*β and peptide·*Ec*β complexes of (**c**) was done by densitometry and plotted as percentage of peptide·*Ec*β bound. Tnp-C WT data is represented with circles and Tnp-C CN is represented as triangles. See Figure S5 for full size gels corresponding to the cropped gels shown in this figure.

To determine whether *Acidiphilium* IS*1634* transposase (Tnp) could bind *Acidiphilium* β, we purified both proteins from overexpressing *E. coli* strains (See Methods) and probed their interaction biochemically. We purified the full-size, wild-type transposase and, for comparative purposes, two engineered mutant proteins, one in which the Q_1_ and the F_5_ of the β-binding motif had been mutated to Alanine (named 5A), and one containing a full consensus sequence (QLSLF, named CN). Also, in addition to *Acidiphilium* β protein (*Ac*β), we purified β from *Leptospirillum ferrooxidans* (*Lf*β) and *Escherichia coli* (*Ec*β) (See Methods). We chose *Leptospirillum* (phylum Nitrospira) because, although distant from *Acidiphilium* phylogenetically, it shares the same acidic habitats, where it is usually the most common organism. *E. coli* was chosen because its β sliding clamp and its ligands have been well described, facilitating the realization of comparative and genetic experiments as described below, and because no IS*1634* member has been yet detected in Enterobacteria. We assayed the interaction by covalently coupling the transposase to tosyl-activated magnetic beads, probing them with fluorescently-labelled β, and extensive washing. As shown in Fig. 3a, wild type Tnp can bind and retain β from *Acidiphilium*, *Leptospirillum* and *Escherichia* but the double mutant (Tnp 5A) could not. The mutant with the consensus sequence motif (CN) could bind to β from the three species.

In order to further demonstrate that the C-terminal regions of Tnp contained a β-binding motif, we synthesized peptides containing these sequences. The peptides, which carried biotin at the N-terminus, were coupled to streptavidin-coated magnetic beads and probed against fluorescently-labelled β from the three species. The results (Fig. 3b), resemble those obtained with full size proteins, and confirm that the C-terminus of the transposase is responsible for the interaction with β.

Since experiments in Fig. 3a,b are mostly qualitative, we used a competition assay to analyze the relative strength of the interaction between WT and CN peptides and *Ec*β. This assay makes use of the gel-shift generated by DNA polymerase IV (LF domain, PolIV^LF^) bound to β in native gel electrophoresis^21^. These two proteins bind strongly and their interaction has been described in atomic detail^27^. PolIV^LF^ binds to *Ec*β at the canonical hydrophobic pocket on the C-side of the ring which is the binding site for all other proteins studied to date. Therefore a displacement of this complex by an excess of peptide, observable in the native gel by a change in the mobility of β, would indicate that the peptide binds strongly to the same pocket on β. A titration of the WT and CN peptides on the preformed complex (See Methods for details) indicate that the CN peptide binds to *Ec*β with higher affinity than the WT peptide, as expected (Fig. 3c,d).

**Figure 4:**
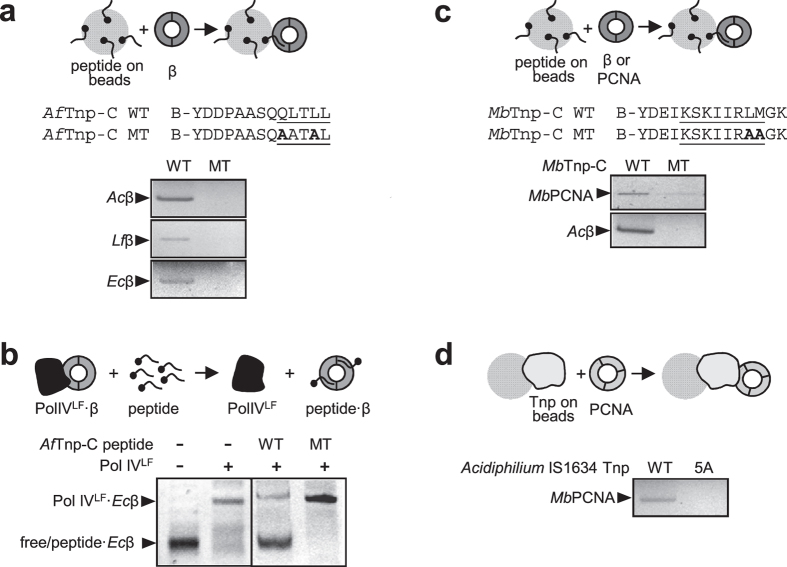
Binding of *Acidithiobacillus* and *Methanosarcina* IS*1634* transposase to sliding clamps. (**a**) Interaction of peptides derived from the *Acidithiobacillus ferrivorans* IS*1634* transposase (WP_014029679) with β from *Acidiphilium*, *Leptospirillum* and *E. coli*. Peptide sequences are shown for the wild-type sequence (WT) and mutant (MT), and the putative β-binding motif underlined. The sequence of the C-terminus of *Acidithiobacillus* transposase starts at residue 4 of the synthetic peptide (see Fig. 2). The biotinylated (B) peptides were bound to streptavidin-coated magnetic beads and assayed with the different β’s. (**b**) Native PAGE was used to separate PolIV^LF^·β complexes from free or peptide-bound β, as in Fig. 3C, demonstrating that the *Af*Tnp-C peptide binds to the same hydrophobic pocket on β as PolIV^LF^. (**c**) Interaction of peptides derived from the *Methanosarcina barkeri* IS*1634* transposase (WP_011306730) with *Methanosarcina barkeri* PCNA and *Acidiphilium* β. Biotinylated peptides were bound to streptavidin-coated magnetic beads and assayed with fluorescently-labelled *Mb*PCNA or *Ac*β. (**d**) Interaction between *Methanosarcina barkeri* PCNA and *Acidiphilium* IS*1634* Tnp (WT and 5A mutant). Wild-type and 5A mutant were coupled to tosyl-activated magnetic beads and probed with labelled *Methanosarcina* PCNA. See Figure S5 for full size gels corresponding to the cropped gels shown in this figure.

The alignment of Fig. 2 showed that in some species (*Aromatoleum*, *Thiomonas*, *Desulfobacca* and *Acidithiobacillus*) a potential second motif can be found at the C-terminal end of some IS*1634* family transposases. To determine whether these sequences could bind to β, we synthesized a peptide containing the C-terminal sequence of *Acidithiobacillus ferrivorans* IS*1634* transposase. As shown in Fig. 4a, this peptide (*Af*Tnp-C WT) can bind and retain *Acidiphilium*, *Leptospirillum* and *E. coli* β, while a double mutant in which the Q and L in first and fourth positions of the motif were mutated cannot (*Af*Tnp-C MT). Further, *Af*Tnp-C WT can displace PolIV^LF^ from a complex with *Ec*β, revealing that the *Acidithiobacillus* peptide binds to the same region of *Ec*β as other peptides (Fig. 4b). Finally, we have also detected a sliding clamp-binding motif in the transposases of two IS*1634* members from Euryarchaea (*Methanosarcina* and *Methanosaeta*) compatible with the consensus PCNA-binding motif (Q-x-x-I-x-x-F-F)^28^. PCNA (Proliferating Cell Nuclear Antigen) is the Archaeal and Eukaryotic sliding clamp, functionally homologous to bacterial β^29^. We purified *Methanosarcina barkeri* PCNA and demonstrated binding to a peptide derived from the IS*1634* transposase present in this organism and containing the putative PCNA binding motif (Fig. 4c). Given the relatedness and overlap between the β and PCNA binding motifs, we also tested whether interactions could be maintained across phylogenetic domains. Certainly, we could observe the interaction between the peptide derived from the *Methanosarcina* transposase and *Acidiphilium* β (Fig. 4c). Then, we tested whether peptides derived from *Acidiphilium* Tnp could interact with *Methanosarcina* PCNA. As shown in Fig. 4d, these proteins interact, showing that the interaction could potentially be maintained, bidirectionally, across the Bacteria-Archaea boundary.

### A stronger β-binding motif increases transposition *in vivo*

**Figure 5:**
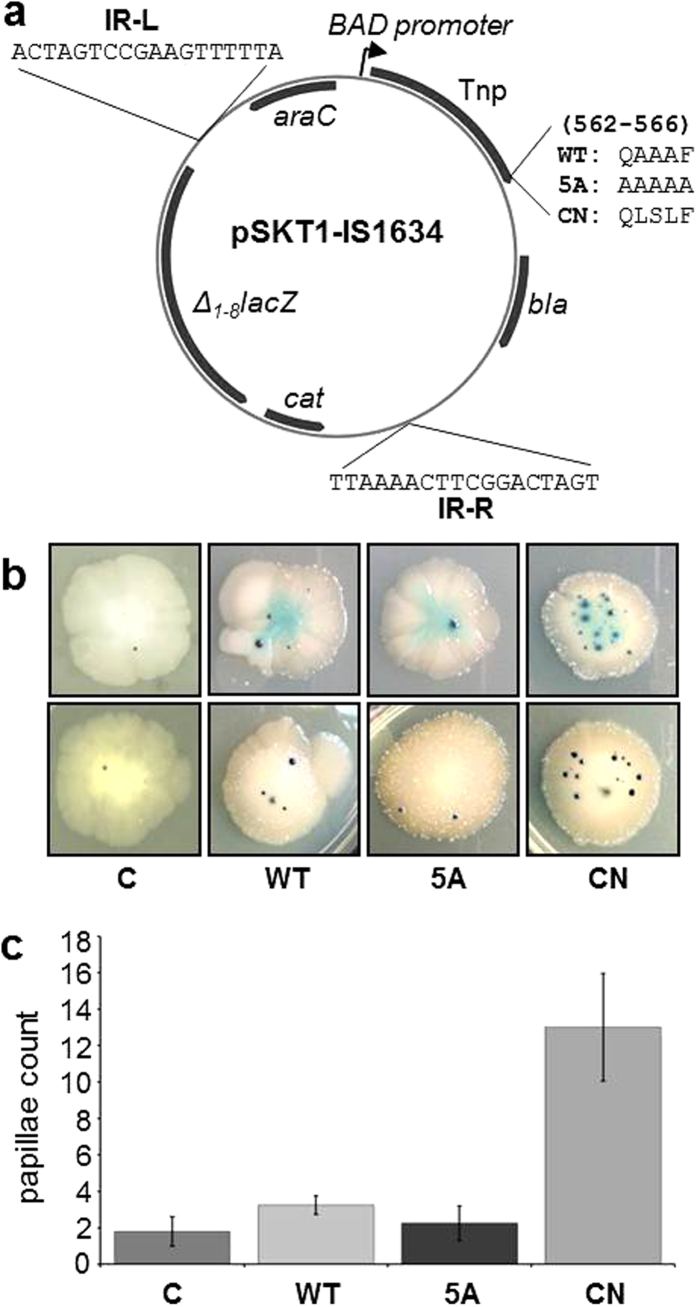
*In vivo* assay to study the effect of different β binding motifs in IS*1634* transposition. (**a**) Plasmid design of vector pSKT1-IS*1634* (See Methods). The sequences for the left (IR-L) or right (IR-R) inverted repeats is shown. The sequences of the wild type and mutants 5A and CN is shown (amino acids 562–566 of IS*1634* Tnp). (**b**) Papillation assay of transposition. The pictures show representative examples of *E. coli* colonies for the three versions of the transposase (WT and 5A or CN mutants), and a negative control C (pSKT1 containing the inverted repeats but no transposase gene). (**c**) Quantification of the papillation assay for 8 colonies (C = 1.51 ± 0.6; WT = 3.15 ± 0.5; 5A = 2.05 ± 0.85; CN = 13.0 ± 2,95).

Although the β-binding motif is universal, the established consensus sequence (QLxLF) was experimentally validated using *Escherichia coli* β^23^,^30^,^31^. A comparison of several β-binding motifs found in *E. coli* and *Acidiphilium* DNA replication and DNA repair enzymes shows that those of *E. coli* better conform to the consensus (Fig. S4). On the other hand, the identity between *Acidiphilium* and *E. coli* β is only 35.4%. Although IS*1634* Tnp binds *E. coli* β (Fig. 3 and crosslinking, Fig. S3), especially the variant containing the β-binding consensus (CN), it was uncertain whether this higher affinity (Fig. 3c,d) would result in more efficient transposition. In order to determine whether the interaction detected *in vitro* with β has an effect in the ability of transposases to be functional in other organisms, we performed an *in vivo* transposition assay for the three variants of the *Acidiphilium* IS*1634* transposase in *E. coli*. We used a recently-developed vector that generates genomic insertions of the *lacZ* gene flanked by the IS inverted repeats^32^. The *Acidiphilium* IS*1634* transposase gene was placed under the transcriptional control of the BAD promoter, allowing for the modulation of its expression by addition of arabinose (Fig. 5a). We transformed *E. coli* DH5α cells with the three plasmid variants containing wild-type Tnp and the mutants 5A and CN, plus a control plasmid with no transposase cloned, and incubated for 15 days at 30 °C. We observed papillae reflecting transposition events in WT colonies, although clearly occurring at a low frequency in our experimental conditions. Mutation 5A showed lower generation of papillae than WT but further experimentation will be required to investigate whether this mutation decreases or eliminates transposition. However, cells harbouring the plasmid with the CN mutation clearly had a significant increase of transposition with respect to WT, generating ~4–6-fold more papillae (Fig. 5b,c). Our results therefore suggest that stronger binding of the transposase to β increases the frequency of transposition events in the cell.

## Discussion

The objective of this study was to identify an active insertion sequence from a natural environment and explore its interaction with the host β sliding clamp regarding its ability to proliferate within the chromosome and its potential for dispersal to other species. We searched for changes in IS abundance in the genome of *Acidiphilium* sp PM that had become fixed in the population during 4 years of laboratory cultivation, and identified one IS that had increased its copy number during this period. We demonstrated that the transposase encoded by this IS interacts with β and mapped the interaction motif to the C-terminus of the protein. Next, we showed that the transposase could bind to β from various species, some of them distant phylogenetically, and identified additional examples of how the interaction between transposases and processivity factors (β and PCNA) can occur even across large phylogenetic distances. Finally, we demonstrated that optimizing the binding site on the transposase to fit the consensus β-binding motif increases transposition in a new host.

Our 600-generation laboratory evolution experiment with *Acidiphilium* sp. PM, aimed at detecting changes in IS copy number that became fixed in the culture by comparative microarray hybridization, found three cases of proliferation and six deletions. Other IS transposition events could have occurred and remained undetected because they were detrimental to the host and were selected against, because they were lost by genetic drift, or because they involved IS relocation in the chromosome (in the case of cut and paste transposition). As it is the case with other studies that have analyzed spontaneous global transposition activity in genomes, our study suggests that only a small fraction of the ISs detected by sequencing are active^33^. The detectable transposition activity can vary greatly with IS element and strain (from 10^−3^ to 10^−7^ per generation, according to some estimates)^34^,^35^,^36^. Unlike the well-defined and relatively predictable point mutation rates resulting from the combined action of DNA polymerases and repair genes, transposition rates are likely the result of the combination of diverse factors such as IS sequence variation, transposase expression and activity, chromosomal location effects, or various host regulatory mechanisms. Additionally, it is likely that the process of isolation and adaptation to laboratory conditions change transposition rates. For example, the observed transposition events in *Ferroplasma* were substantially more frequent in culture than in environmental samples of the same organism^37^. Although transposase gene expression in laboratory conditions has not been explored, metatranscriptomic and metaproteomic analysis of bacterial populations have also detected relatively quick changes in transposase gene expression in response to environmental stimuli^38^,^39^, although the underlying mechanisms are unknown.

The generally low rates of transposition observed in nature suggest that they are determined by regulatory mechanisms that are intrinsic to IS elements, perhaps as a strategy to limit any negative effects on the host genome^9^. However, the interaction of ISs with the essential replication and repair factor β, first demonstrated for transposon Tn7^22^, has now been generalized to nine IS families with very different transposition pathways^21^. This extreme case of functional evolutionary convergence for a highly conserved and universal element suggests that β binding could be an integral aspect of transposition regulation. The β binding motif is present in a large number of proteins involved in DNA synthesis and repair. Since β is limiting in the cell^24^, the concentration of transposases and the strength of their interaction could determine the efficiency of transposition. All proteins so far studied in detail interact with β on the same face of the ring, binding competitively to the same conserved ‘hydrophobic pocket’. Using peptides, it was shown that different types of transposases also bind competitively to the same site on β^21^ and we show here that the same principle applies to the transposase of several members of the IS*1634* family. The affinity with which enzymes bind β is likely to be finely-tuned in the cell to accommodate the various processes for which β is an essential component^24^,^40^. Since β-binding by a transposase could be a burden on the host, it would be unlikely to find strong motifs in transposases. Also, the relatively simplicity of the β binding motif, which often resides in unstructured sequences, could be created *de novo* by random mutation and selection. This could explain the appearance of β motifs at distinct locations in homologous transposases^21^, as it is the case here with the secondary motif identified at the C-terminal sequence of *Acidithiobacillus ferrivorans* IS*1634* (Fig. 4a). Likewise, the fact that the motif appears consistently in non-homologous transposases with very different transposition pathways seems to favour the idea that it has appeared independently many times in the evolutionary history of this diverse group of proteins^21^.

Our results show that interaction with β should not be a strong barrier for IS exchange among phylogenetically-distant but habitat-sharing organisms^3^,^41^. Because ISs are subject to inactivation by mutation, horizontal transfer is an essential aspect of their lifestyle and critical for the persistence of ISs in bacterial populations^2^,^12^. *Acidiphilium* IS*1634* Tnp can bind to *Leptospirillum* or *Escherichia* β, and even to an archaeal sliding clamp, PCNA from *Methanosarcina*. Although β and PCNA share no detectable sequence similarity, these proteins are structurally and functionally very similar, and the binding motifs of the proteins interacting with them are highly related^23^,^24^,^29^. Our results suggest that transposases could interact relatively easily and bidirectionally with the replication machinery of bacterial or archaeal hosts. With a few exceptions (e.g., IS*H6*, found only in Archaea), most IS families can be found in bacterial and archaeal genomes, and genome sequencing suggest that movement of these mobile elements between the two domains is fluid^42^. However, a recent survey of prokaryotic IS elements in eukaryotic genomes detected few events of IS transfer in recent eukaryotic evolutionary history, with the possible exception of cyanobacterial IS*607*^43^. Although HGT events from prokaryotic to eukaryotes have been documented extensively^44^,^45^, it remains to be investigated if mobile elements are involved. In eukaryotic organisms, PCNA-binding has been detected for Pogo, a *Drosophila* transposase^28^,^46^ and, recently, for the endonuclease/reverse transcriptase of LINE-1, where it has been shown to be critical for retrotransposition^47^.

By interacting with β, transposases are targeted to the replication fork, thus associating chromosomal replication with transposition. Coupling of transposition to replication would ensure, first, the possibility of recombination and repair with the sister chromosome, which would limit the potential damage to the host caused by IS excision and the generation of double-strand breaks. Second, IS elements with a conservative (cut and paste) mechanism of transposition would have a chance of jumping to the sister chromosome and therefore increase their number in that replicon. Third, transposition would co-localize with repair factors required in the later stages of transposition to fill in the gaps and ligate (e.g., both DNA polymerase I and DNA ligase interact with β^48^). Yet another possibility is that transposases couple β-binding with allosteric changes that initiate transposition, such as monomer-dimer transitions or binding to the end sequences of the IS. Transposases could require β to be released from a state of self-inhibition which has often been observed in these enzymes^35^. Formation of the transpososome involves dimerization and conformational changes which often involve C-terminal regions of the protein^49^,^50^ which are, almost always, also the sites of interaction with β.

Although transposase binding to β assures integration with the host’s chromosomal replication, it remains to be investigated if an excess of transposase, or a transposase with strong affinity for β, would be disruptive for DNA synthesis. It is remarkable that all enzymes involved in mutagenic processes, namely the DNA polymerases, the mismatch repair system, and transposition, directly interact with the β sliding clamp. Indeed, a recent experimental study demonstrated a direct ‘conflict’ between these processes, as mismatch repair mutator alleles, often present in bacterial populations, limit insertion sequence proliferation in the early stages of invasion in a new host^14^. Further, different IS families could directly compete among them for access to a limiting host resource, the β sliding clamp, as predicted by the genome ecosystem hypothesis^51^. The relation between IS and host genomes has been described as a dynamic equilibrium state, which may become transiently perturbed by the rapid expansion of IS populations and that can be explained just with two parameters: the duplication-deletion ratio and the LGT-deletion ratio^52^. We propose that the ability of transposases to interact with β is one of the molecular processes that contribute to the “duplication” component of the first parameter and, as such, it is subject to natural selection. Variations in the binding site that increase the affinity for β may be selected upon colonization of new hosts and cause explosive proliferation of IS. On the contrary, variations that decrease the affinity for β would reduce transposition rates, contributing to a reduction in IS populations. Future studies will be required to understand how these various processes contribute modulate genetic variability and to the adaptive potential of bacterial populations.

The *in vivo* assay reported here (Fig. 5) shows that transferring IS*1634* from *Acidiphilium* sp. PM to *Escherichia coli* is greatly favoured by point mutations in the motif that make the interaction stronger (Fig. 3c,d). Our IS*1634* transposase CN mutant defines a new category of hyperactive transposases: one with improved interface with the host. The search for hyperactive transposases has been based typically in genetic screens of random point mutants which are then combined in a single transposase to greatly increase its activity. For example, commercial Tn5 transposase contains three amino acid changes which increase activity by >1000-fold^35^, PiggyBac mutations in seven amino acids generate a 17-fold increase in activity^53^, and a combination of multiple mutations in Sleeping Beauty can generate up to a 100-fold increase in transposition^54^. More recent studies have shown that mariner transposases could be easily made hyperactive by mutation of the dimerization interface, thus disrupting the autorregulatory allosteric mechanism between the subunits that limits transposition^55^,^56^. The principles derived from our designed hyperactive IS*1634* mutant could potentially open the door for development of novel hyperactive transposases for use in biotechnology, or for the design of transposases with an expanded range of hosts.

## Methods

### Bacterial strains and long-term cultivation

*Acidiphilium* sp. PM (DSM 24941) was originally isolated from the Tinto river in Huelva (Spain)^57^ and its genome sequenced^25^. The sequenced strain was used to initiate a 100 ml culture in GYE medium, which is composed of a mineral salt solution (0.2% (NH_4_)_2_SO_4_, 0.01% KCl, 0.033% K_2_HPO_4_·3H_2_O, 0.025% MgSO_4_·7H_2_O, 0.0014% Ca(NO_3_)_2_·4H_2_O) supplemented with 0.2% (w/v) glucose and 0.01% (w/v) yeast extract^58^. The pH was adjusted to 2.5 with 1 N H_2_SO_4_ prior to autoclaving (111 °C, 0.5 atm,30 min). Cultivation took place at 30 °C with vigorous shaking. Serial transfers (1:50 dilution) took place in periods of 14 days. Under these conditions the cultures reached stationary phase in 5 days and the culture experienced 9-day-periods in nutrient-deprived medium. The 1:50 dilution allowed ~5.6 generations (log_2_50) per serial dilution, or 584 generations in 4 years. Samples from populations were taken periodically and stored at −80 °C.

### Oligonucleotide microarray of IS-related genes

To design an oligonucleotide microarray that could detect changes in copy number and gene expression of mobile elements in acidophilic organisms, we selected 12 fully sequenced acidophilic genomes (including *Acidiphilium* sp. PM) (manuscript in preparation). We then used RPS Blast (NCBI) to scan 40,231 proteins identified in these genomes with a set of 149 position-specific scoring matrix (PSSM) profiles, selected from Pfam and corresponding to mobile element proteins. This procedure identified a set of 1,358 transposases and associated proteins. The corresponding gene sequences were progressively clustered by means of a customized pipeline that used UCLUST^59^ and Cons^60^ to generate a set of 769 consensus sequences characterized by having at least two conserved blocks of minimal length equal to 50 bp and zero ambiguities. The program Array Designer 4 (Premier Biosoft International) was then used to design two oligonucleotides for each consensus sequence (1,538 oligonucleotides) with an average length of 40 nt and a constant estimated melting temperature (72 °C). See Table S1 for sequences corresponding to *Acidiphilium* IS-related ORFs. Importantly, any IS containing more than one ORF (e.g., one that contains the transposase and an accessory element), will be represented by oligonucleotides from each of these ORFs. In addition to the oligonucleotides representing the mobile genes present in the 12 genomes of acidophiles, we included also three reference genes (rpoB, dnaX and gyrB) for each of these genomes. Spotting was carried out with the MicroGrid-TAS II Arrayer (Genomic Solutions, Huntingdon, UK) at 22 °C and 50–60% relative humidity on epoxy-substrate slides (Arrayit Corp.) according to the manufacturer’s instructions. An array containing 10,752 spots (including three replicas) was constructed.

### qPCR

DNA was extracted from both the initial and final time- points of the long-term *Acidiphilium* sp. pure culture by using Gnome® DNA Insolation Kit (MP Biomedicals). The concentration and quality of extracted DNA was measured by a Qubit® Fluorometer (Life Technologies) according to manufacturer’s recommendations. The primer sequence used in the qPCR were oligo #1 and oligo #2 for the *Acidiphilium* IS*1634* transposase gene; and oligo #3 and #4 for the dnaX gene (See Table S2 for oligonucleotide sequences). PCR reactions (25 μL) were performed with 0.4 μM primers (each), *Acidiphilium* sp. PM ‘2011’ target genomic DNA at two different final concentrations (40 pg/μL and 8 pg/μL) and 12.5 μL of iQ SYBR® Green Supermix (Life Technologies) according to the manufacturer’s instructions. Two biological and three technical replicates were prepared for each gene and each DNA concentration. Reactions were carried out with a MyiQ® Single-Colour Real-Time PCR Detection System (Bio-Rad). Cycling parameters comprised an initial cycle of 3 minutes at 95 °C followed by 40 cycles of 30 seconds at 95 °C, 30 seconds at 60 °C and 30 seconds at 72 °C. A melting curve analysis was performed for each reaction to rule out non specific reaction products or primer dimers. ‘2007’ genomic DNA was serially diluted in 5-fold increments from 5 ng to 1, 6 pg. These serial dilutions with three technical replicates, were used to create a standard curve for the reference dnaX gene (r = 0.992) and another curve for IS*1634* transposase gene (r = 0.997). The genomic copy numbers of *Acidiphilium* IS1634 and dnaX in ‘2011’ time-point was determined from their corresponding ‘2007’ standard curves where both genes are present in a single copy per chromosome, using the comparative threshold cycle method (CT). Results in Fig. 1D represent the average of ‘2011’ dnaX and *Acidiphilium* IS*1634* chromosomal gene copy number and the standard deviation (SD) of the replicates.

### Inverse PCR

Inverse PCR was used to amplify the flanking regions of the *Acidiphilium* IS*1634* insertion sites detected in the ‘2011’ culture. For this purpose 1 μg of ‘2007’ and ‘2011’ *Acidiphilium* sp. genomic DNA, extracted as previously described, were digested with EcoRI (NEB), an enzyme that does not cut within the transposase sequence, for 2 h at 37 °C in 20 μL. Digestions were ligated in 200 μL reaction volume with T4 ligase (NEB) according to the manufacturer’s recommendation. A PCR was performed using 1 μL of ligation products and 1 μM of each divergent primers #5 and #6 (See Table S2). Inverse PCR reaction products of ‘2007’ and ‘2011’ time-points were resolved and visualized in a 1% agarose gel. DNA fragments were extracted of the gel with QIAquick Gel Extraction Kit (QIAGEN) and sequenced.

### Protein purifications

*E. coli* β, *Methanosarcina barkeri* PCNA and GST-PolIV^LF^ were cloned, overexpressed and purified as described^21^. *Acidiphilium* sp. PM β was amplified by PCR using oligonucleotides #7 and #8 (See Table S2), cloned into pET16b (Novagen) as a NcoI/BamHI fragment, and overexpressed in *E. coli* BL21(DE3). 10 g of cells (dry weight) were resuspended in Buffer G (100 mM Tris-HCl, 1M NaCl, 2 mM EDTA, 10% glycerine, 1 mM β-mercaptoethanol, 1 mM PMSF, pH 8) and processed four times with a French press at 4 °C. The lysed cells were diluted to 150 mL and centrifuged (15000 g, 30 m, 4 °C). β was mostly found in pelleted fraction in the form of inclusion bodies. The pellet was washed three times with 20 mL of buffer G + 1% Triton® X-100 (Bio-Rad) to remove cell membranes and residual membrane proteins, and then centrifuged (15000 g, 30 m, 4 °C). The inclusion body pellet was then washed three times with 20 mL of buffer G to remove residual Triton® X-100. In a subsequent step, inclusion bodies were solubilized in 5 mL of buffer H (100 mM Tris-HCl, 100 mM NaCl, 2 mM EDTA, 10% glycerine, 1 mM β-mercaptoethanol, 1 mM PMSF, pH 8) + 6M guanidine·HCl and incubated (25 °C, 15 m). Insoluble material was removed by centrifugation 15 minutes at 15000 g. The solubilised inclusion bodies were slowly drop by drop diluted in 400 ml of buffer H with constant stirring at 4 °C and allowed to refold for 1 hour. The solution was centrifuged 20 minutes at 15000 g to remove insoluble material. This clarified solution was applied on a 30 mL Q sepharose FF (GE Healthcare) ion-exchange chromatography column equilibrated in Buffer H. Protein was eluted with Buffer H over a NaCl gradient (120 mL, 0.1–1.0 M). Fractions containing β were pooled and dialyzed against 2 L of Buffer I (50 mM Tris-HCl, 50 mM NaCl, 1 mM EDTA, 10% glycerine, 1 mM DTT, pH 8). This fraction (1.3 mg mL^−1^) was aliquoted and stored at –80°C.

To amplify the β gene from *Leptospirillum ferrooxidans* by PCR we used oligos #9 and #10 (See Table S2). These oligos introduced a sequence encoding a FLAG epitope at the N-terminus of the gene. The PCR product was cloned as a BglII/NheI fragment into pET11a (Novagen). *L. ferrooxidans* β formed inclusion bodies and was purified following the same protocol as for *Acidiphilium* sp. PM β. The final yield was 1.4 mg mL^−1^.

The gene for the IS*1634* transposase from *Acidiphilium* sp PM (Tnp) was amplified by PCR from genomic DNA with oligonucleotides #11 and #12 (See Table S2), cloned in vector pET16b, and sequenced. The 5A and CN Tnp mutants were created by site-directed mutagenesis of this plasmid by QuickChange Lightning Site-Directed Mutagenesis Kit (Agilent Technologies). Wild-type (WT) and mutant Tnp’s were overexpressed in Rossetta® 2 (DE3) pLysS (Merck Millipore) with 1 mM isopropyl β-D-1-thiogalactopyranoside (IPTG) overnight at 25 °C. After centrifugation, the cell paste was resuspended in Buffer J (50 mM Tris-HCl, 400 mM NaCl, 2 mM EDTA, 10% glycerine, 1 mM β-mercaptoethanol, 0.1% Triton X-100, pH 8) and lysed in a French press. Cells were diluted to 150 mL in the same buffer and centrifuged (15,000 g, 30 min, 4 °C). All three transposases formed inclusion bodies. The pellet was washed three times with 20 mL of buffer J supplemented with 1% Triton X-100, and then washed another three times with 20 mL of buffer J. Afterwards, the pellet was dissolved for 30 minutes at 25 °C in 5 mL of Buffer J + 6M guanidine–HCl, insoluble material was removed by centrifugation (30 m, 15,000 g). This solution was diluted drop by drop in 400 mL of buffer J with constant stirring at 4 °C and allowed to refold for 1 h. Soluble protein was applied to a 15 mL Heparin Sepharose FF (GE Healthcare) ion-exchange chromatography column equilibrated in the same Buffer. Transposases were eluted with Buffer J and a NaCl gradient (120 mL, 0.4–1.0 M), 1.5 mL fractions were collected and those of them containing transposase were dialyzed against 2 L of Buffer J. Tnp was obtained at concentrations of 0.8 mg mL^−1^ for WT; 0.5 mg mL^−1^ for 5A, and 0.5 mgmL-1 for CN.

### *Acidiphilium* IS*1634* Tnp pull-down assay

The binding assay to test the interaction of IS*1634* Tnp (WT, 5A and CN) with β clamp of *Escherichia coli, Acidiphilium sp.* and *Leptospirillum ferrooxidans*, used Dynabeads M-280 Tosyl-activated (Invitrogen). The reactions (50 μL) contained 12 μM of Tnp covalently coupled to 1.2 mg of magnetic beads in Binding Buffer A (phosphate-buffered saline, 0.1% Tween-20, 0.1% BSA, pH 7.2). 5 μM of each β labeled with Alexa Fluor 350 C5-maleimide (Life Technologies) were incubated (15 m, 25 °C) with the Tnp-coated beads, and washed three times with Binding Buffer A to remove unbound β. Reactions were stopped with 1% SDS, subjected to SDS-PAGE electrophoresis and visualized on a UV transilluminator.

### IS*1634* transposase peptides pull-down assay

Peptides were obtained from ProteoGenix SAS (Schiltigheim, France). IS*1634* transposase derived peptides from *Acidiphilium* sp. PM*, Acidithiobacillus ferrivorans* and *Methanosarcina barkeri* were assayed for the interaction with β clamp or PCNA. 400 μM of biotinylated peptides were mixed in 50 μL in Binding Buffer B (50 mM Tris, 50 mM NaCl, 5% glycerine, 0.1% BSA) with 1 mg of Dynabeads M-270 Streptavidin (Invitrogen), incubated (30 m, 25 °C) and washed three times with the same buffer. 4 μM of labeled *Eco*β, *Lf*β, *Ac*β or 3 μM labeled *Mba*PCNA were added to the beads in a reaction volume of 50 μL with Binding Buffer B, incubated (15 m, 25 °C), and washed three times with the same buffer. Reactions were stopped with 1% SDS, loaded on a SDS-PAGE and analyzed on a UV transilluminator.

### Cloning of IS*1634* into pSKT1

The pSKT1 plasmid was generously provided by H. Savilahti (University of Turku, Finland) and used as described^32^. Oligos for cloning of right inverted repeat (RIR) of IS*1634* were #13 and #14 (Table S2); cloning of the left inverted repeat (LIR) were #15 and #16 (Table S2); and cloning of the transposase as a NcoI/EcoRI PCR product were #11 and #17 (Table S2).

## Additional Information

**How to cite this article**: Díaz-Maldonado, H. *et al.* Transposase interaction with the β sliding clamp: effects on insertion sequence proliferation and transposition rate. *Sci. Rep.* **5**, 13329; doi: 10.1038/srep13329 (2015).

## Supplementary Material

Supplementary Information
