# Supplementary material for: Transposase interaction with the β sliding clamp: effects on insertion sequence proliferation and transposition rate
Source: Sci Rep. 2015 Aug 26;5:13329. doi: 10.1038/srep13329 (PMC4549789; doi:10.1038/srep13329)
Supplement: Supplementary Information [file srep13329-s1.doc]

**SUPPLEMENTARY INFORMATION**

**for**

**Transposase interaction with the β sliding clamp: effects on insertion sequence proliferation and transposition rate**

by Héctor Díaz-Maldonado, Manuel J. Gómez, Mercedes Moreno-Paz, Patxi San Martín-Uríz, Ricardo Amils, Víctor Parro & Francisco J. López de Saro

**Table S1** Microarray probes and log_2_(ratio) values for the set of 85 IS-associated genes identified in the *Acidiphilium* sp PM genome

**Table S2** Oligonucleotides used for PCR and other cloning applications

**Figure S1** Structure of IS1634 in *Acidiphilium* sp. PM

**Figure S2** Context of IS1634 in the *Acidiphilium* sp. PM genome (‘2007’ culture)

**Figure S3** Crosslinking of Tnp and β

**Figure S4** β-binding motifs present in enzymes of *E. coli* and *Acidiphilium* sp. PM

**Figure S5** Full-size gels

**Table S1. Microarray probes and log2(ratio) values for the set of 85 IS-associated genes identified in the *Acidiphilium* sp PM genome.** Each IS-related ORF (transposases, accessory proteins) was classified into IS families (See Gómez *et al.*, 2014, for classification scheme). With the exception of five cases, each ORF was represented by two oligonucleotides, and spotted in triplicate in the array. SD, standard deviation.

|  | **IS FAMILY AND ORF NUMBER** | **OLIGO #** | **OLIGONUCLEOTIDE SEQUENCE** | **LOG_2_ RATIO (2011/2007)** | **SD** |
| --- | --- | --- | --- | --- | --- |
| 1 | **IS4-4** | 473  474 | GAGATCCTGGCATTGGAGCGCAGCACGAAGACGCC TGCAGGATCAGCGCGAGAAAAGAACAGAACACATGGC | **-0,022** | 0,301 |
| 2 | **IS4-5** | 383  384 | GCTTCCTCGGGGTTCAGGCAAAGGACATAACGCTTG GGCGTTGATATTCCGAGGTTTTTCAGGATCTCGGC | **-0,530** | 0,444 |
| 3 | **IS66-9** | 1237  1238 | CAACCCCAACTCCATCATAAACCCGAACACAGGTCCCGTC CCGCACTCGCCATTACCCGCACCGGAATGAATTCTGGTAC | **-0,045** | 0,120 |
| 4 | **IS110-1** | 1273  1274 | CATAGATCTTGCCGTGCCGCAACAGAAATCCGGTGAGGTG  CTTCTTCGGAATCAACGATGGCGCAACCACCGTGCAGTCG | **0,295** | 0,064 |
| 5 | **IS110-2** | 1343  1344 | GAATGCTCCGACGGCACAAGCCCCACATAGGCCATCAATT GCATCCTCAACCGCATTGACATAGTCCTGCAGAACGATCT | **0,441** | 0,012 |
| 6 | **IS110-3** | 1231  1232 | CAGGGTCCAGTGCTTCCACGCGCTGAAAACAGTGACAATC GAGATCGAGGACCTGCGGGTAGTATCGCCAAAGTTGCTGA | **0,026** | 0,056 |
| 7 | **IS110-4** | 853  854 | CGTTCTTGTTGCGTTTGACGAAGGGCTTCACATACTGCGG CTCAGCCTCATCGACCCCGTGGATCGTGAAGACATGCTTG | **-0,457** | 0,099 |
| 8 | **IS110-5** | 847  848 | ACTCTCGAAATTTGCCGGGTTTACCGACAGGGCTATGGTA GATCTTCTGATCGAGTTCAGCGACATGCCGTCCCATCTGG | **0,440** | 0,007 |
| 9 | **IS110-6** | 849  850 | GACATTTCCTTTGCCTTTGGCCGCGACGATACCGAACTCC TTTGACGAAAGGCTTCACGTATTGCGGCGGGATCAGCTTC | **0,112** | 0,045 |
| 10 | **IS110-7** | 181  182 | CTGATTGCTTCGGCATCATTTGCGTCGTTCTTCTGGTTGC GATAATCAGCAAACGCCGCAAGGTTCGCTCGCCCATCTTC | **0,300** | 0,021 |
| 11 | **IS110-8** | 1235  1236 | CTTCACGAAGGGCTTCACATATTGCGGTGGGATCAACCTG TTCCTCGGCAGACAGGAGAACAAGCAATGCTGAGACGTTC | **0,260** | 0,014 |
| 12 | **IS110-9** | 161  162 | CAACTGTCCGGTCTCGAGCCCGACCCGAAC CCCTCTTGCCCCGCCAGACCAGCTTCCCGT | **0,302** | 0,184 |
| 13 | **IS110-10** | 1323  1324 | CAAACAGCCAAGCGGTCGCTGATTTGCTGCCTGGTTTAGC TCATTGCCTGCCTTACTTATCTTTCCCATACGATGCTTGCCG | **0,250** | 0,134 |
| 14 | **IS110-11** | 23  24 | CAAATAACGATTGCCGCGTTTGCTGATGCCGAGCAG CGCCAAAACGGCCCATGCGATCCGAGCCAGCTTCG | **0,040** | 0,071 |
| 15 | **IS110-12** | 1327  1328 | GTCGTTCCTTCTCGTCCACCGCGTGGATCGTGAAAACATG GTGAGTGACCAGCTCGAAATCTGGATGGATCAGCTTCACC | **-0,415** | 0,092 |
| 16 | **IS1380-1** | 145  146 | CGAGCGTTTCAGGGTTTCGAGGCGGACGGCGAGTTTGATC CGCCATCATTGAACTCTTTGAACCGCCGGATTTTCTCTCC | **0,213** | 0,222 |
| 17 | **IS1595-1** | 901  902 | GAAATCCTTGAGGCGGGAATGGCGGTTATTGACGGTCTGG GTTGGGATGAAGGCCGATGAGGTGAAACCAGCGAAGATAG | **0,136** | 0,120 |
| 18 | **IS1634-1** | 535  536 | AGCACCGGCCTTCAAGATAGGACGAGCTGACATCATAGAG CCACTCGACGTAATAGGCAATCATGCAGAGAAAGACATGCG | **1,612** | 0,046 |
| 19 | **IS21-1** | 239  240 | ACGGGATCGCGGTGAAGTCGAAGGTATCGAGGCTT CGGTGCCGGAATTTCCCAGAGCGATGATGTTGTCG | **-0,563** | 1,582 |
| 20 | **IS21-2** | 1436 | GATCGAGGAGTGCGGTGGTCATCTTGGCATCGCCGAAGAC | **0,299** | 0,100 |
| 21 | **IS21-3** | 1021 | CAAGAGCATTGACCAGATCGACGGTGGCAAATAACCTGAT | **-0,050** | 0,130 |
| 22 | **IS256-1** | 1295  1296 | CTGTTCTCCGCCTCCGCTTCGTCACTCAGATGGTAATCCA TCTTCGCAATAAAAGGTAGAGGCTTGACCCACATTTCTCACGC | **0,334** | 0,066 |
| 23 | **IS256-2** | 363  364 | CATCCCCCGCGCATACATCGAGATGATCTTGTCATCGAAG CTGTTCTCCGCCTCCGCTTCGTCACTCAGATGGTAATCCA | **0,352** | 0,201 |
| 24 | **IS256-3** | 951  952 | CTCGCGCACCTTCACATAGGTGGCATCGAGCCAGAGATAG GTTGGGGAAGATACAAACGACATCGGCACGGCGCTTGATC | **0,016** | 0,008 |
| 25 | **IS256-4** | 1475  1476 | CCGGTCAGCGCCTTGACTTCCGTCTCCATGATCCGTTCGG CTTCTCAACCAGCTCGATCAGCGCCATTCTCTCGTCGGTC | **-0,133** | 0,137 |
| 26 | **IS256-5** | 977  978 | CATGGATTTCGGAAAGTGGTTGAGCACGTTGGCGATCTTG GTAGTCATAGAAGGCCAGCAGAACCTCGGTGTCCTTGGTC | **0,385** | 0,098 |
| 27 | **IS256-6** | 113  114 | CTTGAGATAGGTCGCATCCAACCACAGATACGGCCACTCG GATCAGCCCATCCACATCCGTCTCCATCAGCAGTTGAACC | **0,395** | 0,219 |
| 28 | **IS3-1** | 1107  1108 | CAAAACGACCTGAACGACTTCCGCTGACCAAAATAAGCCC CACTATGCCACTCACACACGAAATTCAGGACACTGCCGTC | **0,295** | 0,375 |
| 29 | **IS3-2** | 415  416 | CTTCCACAAAATCGTAGGACCACACATGGCCGGGATACTC TGATGTAGAGCGTCTTCACACCGACCTTCCCGAGCCATTG | **0,132** | 0,118 |
| 30 | **IS3-3** | 1203  1204 | CGGGCGATGAATTCGGGACCATTATCCGACCGTATATGCG GTTGAGCAGCTTATCACGCAGCGAGCCATTGAAGGATTCG | **0,109** | 0,078 |
| 31 | **IS3-4** | 241  242 | CCCCGAACTTTCATCCGTGTGCGATTGGAGTCTCG GGCATGATCGAGGCCGAAGAACAGGCTCTCGTTCA | **0,289** | 0,155 |
| 32 | **IS3-5** | 1269  1270 | CTTCCGCTTCCGCCACAATCCGTAGCTTGTCCTCATCACG TATCTTCAGCCCCTGCAATCATCACGGCAGCAGACGGCTC | **0,540** | 0,026 |
| 33 | **IS3-6** | 1379  1380 | CCGTTGATGGTTTCAGAGAGGGCATTGTCATAGGAATCGC CGAGGAAGAGGCGGTTGTTGATCCAGTCCACCCATTCGAG | **0,346** | 0,078 |
| 34 | **IS3-7** | 959  960 | ACTTCATCTGGTCGATAGCGGCGAAATATCGTTGTTCGGC ATCAGGCGTTCGGTGTATTTGATCGAGACATACTGGCTGC | **0,132** | 0,086 |
| 35 | **IS3-8** | 1497  1498 | CAAGCGGCGATCTTCATCTGGTCGAGTGTGGCGAAATATC GAGAAGGCGTCTGTGGTTGAGCCAGTCGACCCATTCGAGG | **0,307** | 0,303 |
| 36 | **IS3-9** | 265  266 | GATACGCCGGGCGAATGTATCGATGACGAACGCCA TCGTGAAGAGGCTGTTCCAGGGCATCGAGGACGAA | **0,157** | 0,174 |
| 37 | **IS3-10** | 1299  1300 | CTTCAGCTTCGCAGATACTTCCGACGGAACACCAGGCGCA GGACTTCGGCAGAAAATTTGTTCGTCGTCGGACTCGTCAT | **0,198** | 0,045 |
| 38 | **IS3-11** | 1103  1104 | CGGCAGGTGGTTCAGCAGGTCTTCATAGGCATTGATAAAGG GTACCAGGTTCGGATCGTGTCATCATCCAGCAGCAGCACC | **-0,480** | 0,033 |
| 39 | **IS3-12** | 1411  1412 | AGACCTTGCGGACACCATAGACCTGGAAATTCTCTTCCCA GTTTGGGTCAGTACGACGTGCGGCATGCTCATAGTAGGTC | **0,019** | 0,064 |
| 40 | **IS3-13** | 1417  1418 | CAACTTCAGGCCGCCATACTCCTTGCGCCACCGATAATAG GAACAATCTCAGCCTCGCGCAACTTGCCGATGATCTCTTC | **0,207** | 0,217 |
| 41 | **IS4-1** | 73  74 | GCAGAAGAAATTCTCGATCAGATGACGCCATTTGTAGGTTTCGTGG TTGCTGTCGAATGCCTTGTCTGCGATCAATCCACCGAATT | **0,358** | 0,007 |
| 42 | **IS4-2** | 201  202 | CGTCAGCCTGGTGAAAGATCGGCCTTGTATCGAAGCTGGC CTGGAGCCTGTCGAGATCGTTCAGCAACTTCTGCCATTCG | **0,289** | 0,074 |
| 43 | **IS4-3** | 1133  1134 | GAAATACAGCGAGGTGGTGTCCATGAACACGACCGAGAGG GTGCCGGGAATGTCGTAATCGTCGAGCCATTTGTCGCAGG | **0,200** | 0,048 |
| 44 | **ISAs1-1** | 89  90 | CAGGATTTCGGGTTGATCGTCGCTGCTGGACAGGAAGTAG GAAACGCCCGGTATCGATCAGCATGAACACACGTCGGAAG | **-0,150** | 0,014 |
| 45 | **IS5b-7** | 1347  1348 | CTCACCACTCTCGGTCGTCTTGACGCTCTGACTGTCGATC GAACTGTCTGTCGGCCTTTCGCACCACCTCGATGCGGATT | **0,245** | 0,077 |
| 46 | **IS5b-8** | 1349  1350 | CAGGAAGTTGGCGGCCTTCTTATCGTAGCGAGTTGCGATG CCAGATAGTCTCTGATGTCGTCTGCGTCATAGGCTTTGTCG | **0,704** | 0,005 |
| 47 | **IS5b-9** | 1407  1408 | GAGGAAGTTTCGTGCGGTCTTGTCGTAACGTGTAGCGATG TCATCGGCGTCGTAGGCCTTGTCGGCGATCAGGTCTTCTG | **0,400** | 0,010 |
| 48 | **IS5a-1** | 973 | GGGGTGGATGGTATTGGGGAGATTCGGAGGGCTTATTTTG | **0,304** | 0,130 |
| 49 | **IS5a-2** | 227  228 | ATCGCTTCGTCGGACAGCCCATCCATGTGCTTGAGAAGAT GGAACAGTTCGAGATCATCCTGCGAGGCATTCAGCTTCGG | **-0,526** | 0,127 |
| 50 | **IS5a-3** | 1233  1234 | CTCGATGAGATTGCGCTTTCTGTAGGCGACGGGGTCGAAG CTGATCCATGAGGTGACGGCGAGGACGAGACAGATACCTG | **0,133** | 0,081 |
| 51 | **IS5b-1** | 1001  1002 | CCGCAACCAAGGATACAGGGATTTGACGGATTTGAGCACG TCGACAGCATGTAGAAGATGGCCTCCACAATCCTTCGCAG | **0,190** | 0,127 |
| 52 | **IS5b-2** | 1515  1516 | CTGCCCATTGCGTGTCGTTCAGCCAGAATAGCTCC GAGCCCTCGCGAAAGCGATGCAGGATGCCGCTCAG | **0,295** | 0,115 |
| 53 | **IS5b-3** | 1117  1118 | CGTCGAGATCGGTCTTGAGGTGAATTTTGGTCGGGAAGCC TACAACCGCTTCGGGAAGAATTGGCCTCTTTCCTTGGAATTT | **0,377** | 0,222 |
| 54 | **IS5b-4** | 1479  1480 | AATCCCGCCTCGCAGGACGTAGAAGATCGCGTTGACAATC CAGATGATGGTTGATCCGCTCCCAGGTGCCATTGTCACGG | **0,014** | 0,020 |
| 55 | **IS5b-5** | 1131  1132 | GAACACGTCAAGGCGGCTCCAGCGGATAAACTGATTGTAG GTGAGGCAGCGGCTCTTTCCCTCTCCTGATTGATGGAATG | **0,131** | 0,086 |
| 56 | **IS5b-6** | 441  442 | CGCTTGACGATCTGTATGGTCCATTTGCCGATTTTGGTGAG GTGGTGAACGGTGCGAATTCCTTCGGGATCTGCCACCTCC | **0,315** | 0,007 |
| 57 | **IS6-1** | 1205  1206 | CCGATGGTCCTGCTCAACGAGGTTATTCAGATACTTCGACGAT CGGTAGAGATAGACCCATTCACCCTGGACATTCACGTAGGT | **0,019** | 0,225 |
| 58 | **IS6-2** | 1389  1390 | ACGCTTGGGGCTGAGACGGAAGTCAACGGCATTTC CTTCACGTAGGTCTCGTCCACACGCCACGACGCTC | **0,118** | 0,116 |
| 59 | **IS6-3** | 1535  1536 | CATCGACAGGCCGCGCTCCTCCATCATCTCCAC GTTCCACCGGCGCTCGAATTCCGGCGAATAGTG | **0,200** | 0,005 |
| 60 | **IS630-1** | 1337 | AAATTGTCCATGATCACGAGATCGCCCGGCGTCAGGGTTG | **0,270** | 0,100 |
| 61 | **IS630-2** | 1239  1240 | AATCGGGTTGAAATCCGGCGAGTATGGCGGGATGAACAAG GTTTTCCGACCGTGTCCCAGAGTGCGTTGATCGTTCTTTC | **0,385** | 0,064 |
| 62 | **IS630-3** | 1285  1286 | CAACCACCCGTTCACGCAAATCCAGCGAAAGTACCTTCAC GAAGAACCGCCAGATCGTCGAAATGCCAAACCACTCCGAA | **-0,001** | 0,219 |
| 63 | **IS630-4** | 1145  1146 | GATGATGGGTGCTTTGTGGCTGCCGAGATTGTCCATGATC TCAACATGATGGTGTCGATGATCTGTCCGATTGCACTCCAG | **0,004** | 0,023 |
| 64 | **IS630-5** | 1229  1230 | GAAGCTCGGGAATGAGGACGTACTCAACCCAGGTCTCGAA GGTTGAAGTCTGGGCTGTAGGGCGGCAGATATCGTAACTC | **0,154** | 0,014 |
| 65 | **IS630-6** | 783  784 | ACATTCTTCTCAACCATGTCCAGGAAGCGGCGGAAGTCTG TTCTCCAGCGTCGTCGTTATCACCTCGGCAATCTTGTCAT | **-0,070** | 0,297 |
| 66 | **IS630-7** | 108 | GACATATGCCTCGAACCAGTCGCCGTTGATGGGGCCGTCC | **0,293** | 0,130 |
| 67 | **IS630-8** | 1405  1406 | GATCGGGTTAAAGTCGGGGCTGTAGGGTGGAAGATAGAGC GGTGATGGTGTCGATGATCTGTCCGATTGCACTCCAGAGG | **-0,006** | 0,020 |
| 68 | **IS66-1** | 1503  1504 | GAAACCCCACCTTCGCGTCGTCGTTAATTACGTCCA GGCAACAATCCGCGTCTTCTCCTCGTCCGACCATC | **0,193** | 0,113 |
| 69 | **IS66-2** | 561  562 | CACATTGCTTCTCCTCAAGATCGACCACGACCTCGACCTG GAGTTTTCCAGTTCCAAGGCAGCAGGGTGTCGATTTCGTT | **0,321** | 0,149 |
| 70 | **IS66-3** | 611  612 | CGTCTTTCTCAGCGTGCGATAGGAGAGACAGGTCAGGAAG GGCGATATCCGGGTGGTCGAGGAAGGTGAAGAGATGATCA | **0,509** | 0,142 |
| 71 | **IS66-4** | 1509  1510 | CAAGATAGACCCGGACACCCGCACCCGGCGCGATC GTTCCCGCCAAAGACCTGCTGGACCATCATCGCCA | **0,847** | 0,311 |
| 72 | **IS66-5** | 1339  1340 | CCAGATAAACCGCCCTCGATCAAGGCGTTTCGAATACAGC GGATTTCGCCAGTCGATCCCCTCCAGAAGATAACCGATCT | **-0,065** | 0,049 |
| 73 | **IS66-6** | 1089  1090 | CTTCAGCCAGATCGCCATCCCGATATTGCCGACGC GACACCGTCGTGCCAGAGCAGCTTGATCTGTCCAG | **0,205** | 0,205 |
| 74 | **IS66-7** | 263  264 | CATGCCAGATGATCTTCACCAGCGAGCCCGACCTG GCTGCGAACTCGTCAACGAAACCACCCCGTCCTTC | **0,256** | 0,185 |
| 75 | **IS66-8** | 307  308 | CGGAGCATGTAGTTGATCGCCTTGGCAAGATCATGGTGGC GTTATTGAGGCGGGCGGTCTGGATCAGGGTGTAGAGGATG | **0,262** | 0,066 |
| 76 | **IS701-1** | 273  274 | GCCCGATCGATAAAGGCATGGCATGAGCCGACA GGCCGTTGGCAATCTTGCCCGCCGAGCCGG | **0,310** | 0,092 |
| 77 | **IS701-2** | 687  688 | CAATGTATCCTCAACCGACGCACCGCCGATCATCATGTCC GGAGTGTCATTTCAATCTCGCCCACGCCGTAGACGCTATC | **0,478** | 0,110 |
| 78 | **IS701-3** | 679  680 | CCTCGTCGCCAGTTGATCCTTCGCCACTTCTCTTCAATCA GACTGACAATTCGCCGTCTTGCCCAACGCAGAAGCATATT | **0,546** | 0,003 |
| 79 | **ISL3-1** | 1223  1224 | GCATACATCGAGATGATCTTGTCATCGAAGCCCGGAAACCG TGTCGTGGGCGTTGATGATTCGGCTTGGCGGCGTAATTTC | **0,319** | 0,326 |
| 80 | **ISL3-2** | 119  120 | GAAAGAAGTTGAGGTGCCGCCAGCTCAGTTCCTTCCAGTC GCATCGAGGTCAATGAACACGGTGACGTAGCTGTGGCCTC | **0,278** | 0,158 |
| 81 | **ISL3-3** | 1143  1144 | CAAGATATCACAGACGAGTTTGCGACTATGGCCGGTGCCG CGGCATTCAGGAAGGCGTGGCTGGCATTCTCCATGAGATG | **0,027** | 0,057 |
| 82 | **Tn3-1** | 1209  1210 | GTGGGTCCAAGACCAAATTCCTTCAGCCGCTTGACCTCAG TCAGCTCGTGAACGATATCCTGAAGGTCCTTCATCGACCG | **0,278** | 0,219 |
| 83 | **Tn3-2** | 291  292 | GATCGGAGAGATGGGTGTAGAATTTCAGACCCGGTTCGGG GCATAATGTTCGCTCAGCACCTCGTAGGGATCGAAATCTTCC | **0,043** | 0,215 |
| 84 | **Tn3-3** | 253  254 | GAAATCGTGCTGTGGCTGACGTTGTAGGAGCGGGC GGCCTTCGCCCGTTCTCTTCCTTCTCCGGTCCGGG | **0,000** | 0,061 |
| 85 | **Tn3-4** | 923  924 | CAAACCTGTAACCCAGCAGATGGCAGAGACCGAAGACATG GATAGATGGTATTCCAGTGGACGATGGCGGCGGTGATGAG | **-0,025** | 0,095 |

**Table S2. List of oligonucleotides used for PCR and cloning.**

| 1 | 5'-AAGGCACGATCGCCAATCTGA-3’ |
| --- | --- |
| 2 | 5'-CAGCAGGCGCGGAATCTCCAG-3’ |
| 3 | 5'-CGTCCTCGCCCGCAAATACC-3’ |
| 4 | 5'-AATCCGCGCCGTCGTCGTCTT-3’ |
| 5 | 5’-GCTCTCAAGCTTTGCCGACCTGATGGCGCATC-3’ |
| 6 | 5’-GCCATCGACCAGCTCCGCCGGCATCTGGCTCAG-3’ |
| 7 | 5’-CTGGTCCCATGGCGCTCAAGGCGGATCGGGCAACG-3’ |
| 8 | 5’-CTGGTCGGATCCTCAGACCCGCATCGGCATGAGAACGAAAATCG-3’ |
| 9 | 5’-CGGCTAGCTAGCGACTACAAGGACGACGATGACAAAGAAATTATCGTTGATCAGGAGAC-3’ |
| 10 | 5’-GGCCTAAGATCTTCACCCCATGGGCATTAACACATACCGGG-3’ |
| 11 | 5’-GCGAACCATGGTCATCGACGTGGTTCCGAATGGCC-3’ |
| 12 | 5’-GCCATACCCATATGCTACTGGACACGCATGGGTTCG-3’ |
| 13 | 5’-GGCCGTTAAAACTTCGGACTAGTACTCGAG-3’ |
| 14 | 5’-CTAGCTCGAGTACTAGTCCGAAGTTTTAAC-3’ |
| 15 | 5’-CTAGCTCGAGGACTAGTCCGAAGTTTTTAT-3’ |
| 16 | 5’-CCGGATAAAAACTTCGGACTAGTCCTCGAG-3’ |
| 17 | 5’-GCTACCGAATTCCTACTGGACACGCATGGGTTCG-3’ |

**A**

**ccgaagttttta**tttgaaaatcatctaaacgtgaggctccgtgcgggatacggctcgttattaatttcagaatgtgtccatacatggggcttgcggcgcgactcgatttgtgattcgctggcgtcatgttcatcgacgtggttccgaatggccgctcggcgtcggcggtgctgctgcgggagagtttccgtgaggggcggaaggtccacaagcgcacgatcgccaatctgagccagatgccggcggagctggtcgatggcttgcgcgccctgctcgccggcggctcggtggtgggcggcccggatcaggcgctcgagatccggcgatccctgccgcacgggcacgtggcggcggtgctggggatgatgcgcaagctggagattccgcgcctgctgggacgccaggtctcgcgcgagcgggacttggcactggcactgatcgcgagtcgcgtgatcgcgccgggctcgaaactctcgacgctgcgcggcctgaacccggagacggcgacctcgagcctcgggcaggtgcttgggctcggcgtgattgaggagcgcgagatctacgccgccctcgactggttgggcgcgcagcaggggcggatcgaacggcagttcgcgaagcgccatctgcgcgatggcacgctggtgctctatgatgtcagctcgtcctatcttgaaggccggtgctgcgaactcgctcaacacggttatagccgtgatcatcggccggatcggctccagatcgtctatggtctgctgtgcgatcgggagggccggccgatcgcggtcgaggtgttcgaaggcaacaccgccgatcccggcacgatcgcggcccaggtggagaagctcaagcggcggtttcatctgaaccacgtcgtgctggtgggcgatcggggcatgatcaccacggcgcggatccgcaaggagatcaaacccgccggattggactggatcagttgcctgcgggcgggtcagatccaggacctcgccgaggggccgctgcagatgtcgctgttcgacgagcgcgatatcgccgcgatcgcatcgcctgattatcccggcgagcggctgatcgcctgccgcaatgcggctctggccggggaacgccggcgcaagcgcgaggcgctgcttaccgcgaccgagcgggaattgacccgcatcgtggcggcgacgacgcgcaagcgcgcgccgttgcgcggcgcggccgagatcggccttgcggtcggtgccgtgatcaaccagcgcaagatggccaagcatttcgatctcaccatcaccgccgatcgcttcagcttccggcgcaacgaagccggtatcgcccgcgaggcggcgctcgatggcatttacgtcatccggaccagcgtcgccgcggaggcgatgagcgatgccgacaccgtgcgggcctacaaggacctctcccgggtggaacgggcgttccgaaccctgaaatcggtcgacctcgcaatccgtccggtccatcactggctctcgccgcgggtgcgcgcgcatgtctttctctgcatgattgcctattacgtcgagtggcatctgcgcgatgccctcaagccgatcctgtttcaggatcacgatccactggccgccgaggccgagcgcgcctccccggttgcccctgccacgatctcacccgccgccaagcgcaagcgggggcgacgccgcaacgacgacaacctgccgctctcaagctttgccgacctgatggcgcatctggccacccaaactctcaacaccgccgcgctgcccaaggcgcccaatgcgaccttcaccaccctggccacgccaaccacactacaggcggccgccttcaacctcctcgaaatcgaacccatgcgtgtccagtagacgtcaaaagcggatcaaaaaatccgccgataaatccagggactttgtattccgcctag**ttaaaacttcgg**

**B**

10 20 30 40 50 60
MFIDVVPNGR SASAVLLRES FREGRKVHKR TIANLSQMPA ELVDGLRALL AGGSVVGGPD

 70 80 90 100 110 120
QALEIRRSLP HGHVAAVLGM MRKLEIPRLL GRQVSRERDL ALALIASRVI APGSKLSTLR

 130 140 150 160 170 180
GLNPETATSS LGQVLGLGVI EEREIYAALD WLGAQQGRIE RQFAKRHLRD GTLVLY**D**VSS
 *****
 190 200 210 220 230 240
SYLEGRCCEL AQHGYSRDHR PDRLQIVYGL LCDREGRPIA VEVFEGNTAD PGTIAAQVEK

 250 260 270 280 290 300
LKRRFHLNHV VLVG**D**RGMIT TARIRKEIKP AGLDWISCLR AGQIQDLAEG PLQMSLFDER
 *****
 310 320 330 340 350 360
DIAAIASPDY PGERLIACRN AALAGERRRK REALLTATER ELTRIVAATT RKRAPLRGAA

 370 380 390 400 410 420
EIGLAVGAVI NQRKMAKHFD LTITADRFSF RRNEAGIARE AALDGIYVIR TSVAAEAMSD

 430 440 450 460 470 480
ADTVRAYKDL SRV**E**RAFRTL **K**SVDLAIRPV HHWLSPRVRA HVFLCMIAYY VEWHLRDALK
 ***** *****
 490 500 510 520 530 540
PILFQDHDPL AAEAERASPV APATISPAAK RKRGRRRNDD NLPLSSFADL MAHLATQTLN

 550 560 570
TAALPKAPNA TFTTLATPTT L**QAAAF**NLLE IEPMRVQ

**Figure S1. Structure of IS1634 in *Acidiphilium* sp. PM.** A. The insertion sequence (1930 bp) contains 12-bp-long inverted repeats (bold). The initiation and stop codons of the transposase gene are boxed and shaded. B. Sequence of the IS1634 Tnp. The putative DDE motif is marked with asterisks and the β-binding motif is boxed.


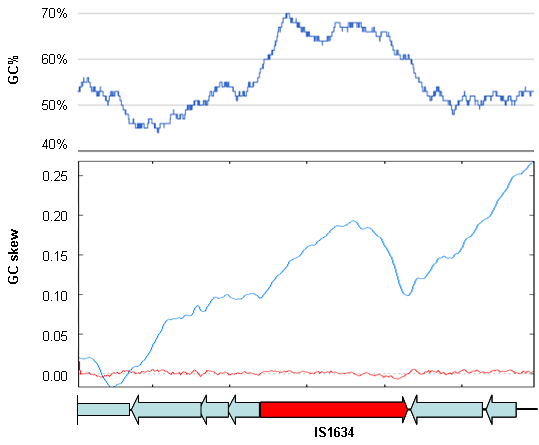


**Figure S2. Context of IS1634 in the *Acidiphilium* sp. PM genome.** Top panel**,** the content of G+C was calculated in the genomic region containing IS1634 using the program GC content calculator (www.biologicscorp.com) with a window of 500. Bottom panel, the cumulative GC skew (blue line), [(G - C)/(G + C)] was calculated using online GC skewing software (http://gcat.davidson.edu) with a window of 100 and a step size of 20. The region spans a region of 5986 bp.


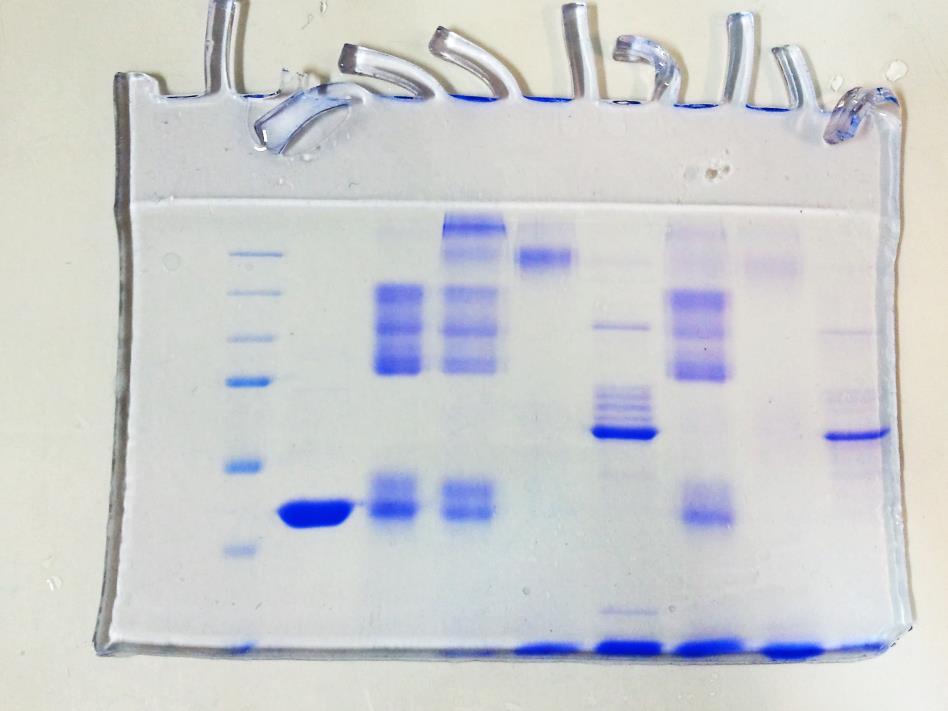


Tnp_4_-β_4_

β_1_

Tnp_1_

Tnp_4_

Tnp_2_

*

1 2 3 4 5

250

150

100

75

50

37

443

MW (kDa,

x1000)

BS(PEG)5

Tnp

**- - + + +**

*Ec*β

**+ + + - -**

**- + + + -**

**Figure S3. Chemical crosslinking of Tnp and β.** SDS-PAGE of *Ec*β and *Acidiphilium* IS1634 Tnp. In lanes 1 and 5, β and Tnp, respectively, migrate according to their expected molecular weights in the absence of the crosslinker (β, 40 kDa; Tnp, 64 kDa). BS(PEG)5 crosslinking reveals that β readily crosslinks in dimers (80 kDa), trimers (120 kDa) and tetramers (160 kDa) (lane 2, shown with an asterisk). In the presence of BS(PEG)5, *Acidiphilium* IS1634 Tnp crosslinks into a form that is consistent with a tetrameric transposase (254 kDa, lane 4). When β and Tnp are mixed in the presence of crosslinker (lane3) there is a new complex (~414 kDa) formed which is consistent with tetrameric Tnp and two dimers of β (Tnp_4_-β_4_).

Reactions were performed in 20 µl in phosphate-buffered saline pH 7.2 and supplemented as indicated with *Escherichia coli* β (4 µM), IS1634 Tnp (4 µM), and 0.5 mM BS(PEG)5 crosslinker (Pierce Biotechnology), and incubated (30 m, 25°C). The reactions were quenched by incubation with 50 mM Tris-HCl, pH 7.5 (15 m, 25 °C). Products were analyzed by 8%-polyacrilamide SDS-PAGE and visualized by Coomassie staining.

**Pol III (α) a** *Ec* -G**Q**AD**MF**G-

*Ac* -G**QI**N**LF**G-

**Pol III (α) b** *Ec* -E**QV**E**L**E**F**D*

*Ac* -GVAE**V**AEL*

**Pol III (ε)** *Ec* -G**Q**TS**M**A**F**A-

*Ac* -R**Q**RG**L**DLA-

**Pol III (δ)**  *Ec* -C**Q**AMS**LF**A-

*Ac* -E**Q**ADR**L**PE-

**UmuC** *Ec* -A**QL**N**LF**D-

*Ac* -R**Q**AV**LF**A-

**MutS** *Ec* -T**QM**S**LL**S-

*Ac* -D**QLS**PRA-

**MutL** *Ec* -A**Q**PL**LI**P-

*Ac* -A**Q**AL**L**AP-

**Figure S4. β-binding motifs present in enzymes of *E. coli* (*Ec*) and *Acidiphilium* sp. PM (*Ac*).** Residues shown correspond to the β-binding motifs present on the α (a: internal; b: C-terminal), ε and δ subunits of DNA polymerase III; UmuC (DNA polymerase V); and the mismatch repair enzymes MutS and MutL. These enzymes were fully aligned but only the section corresponding to the β-binding motifs is shown.

**Figure S5. Full-size gels corresponding to those of Figures 3 and 4, as indicated.** In all cases the protein band observed corresponds to purified and labelled (alexa 350) *Acidiphilium* (Ac), *Leptospirillum* (Lf) or *E. coli* (Ec) β, or to *Methanosarcina* PCNA (Mb).
